# Floral Volatile Organic Compounds Change the Composition and Function of the Endophytic Fungal Community in the Flowers of *Osmanthus fragrans*

**DOI:** 10.3390/ijms25020857

**Published:** 2024-01-10

**Authors:** Tingting Shi, Man Shi, Yunfang Ye, Yuanzheng Yue, Lianggui Wang, Xiulian Yang

**Affiliations:** 1 Key Laboratory of Landscape Architecture, Jiangsu Province, College of Landscape Architecture, Nanjing Forestry University, Nanjing 210037, China; tingtingshi@njfu.edu.cn (T.S.); yeyunfang2021@163.com (Y.Y.); yueyuanzheng@njfu.edu.cn (Y.Y.); wlg@njfu.com.cn (L.W.); 2 State Key Laboratory of Subtropical Silviculture, Zhejiang A&F University, Hangzhou 311300, China

**Keywords:** *Osmanthus* *fragrans*, volatile organic compounds, endophytic fungi, community composition, function prediction

## Abstract

Endophytic fungi in flowers influence plant health and reproduction. However, whether floral volatile organic compounds (VOCs) affect the composition and function of the endophytic fungal community remains unclear. Here, gas chromatography–mass spectrometry (GC–MS) and high-throughput sequencing were used to explore the relationship between floral VOCs and the endophytic fungal community during different flower development stages in *Osmanthus fragrans* ‘Rixiang Gui’. The results showed that the composition of the endophytic fungal community and floral VOCs shifted along with flowering development. The highest and lowest α diversity of the endophytic fungal community occurred in the flower fading stage and full blooming stage, respectively. The dominant fungi, including Dothideomycetes (class), Pleosporales (order), and *Neocladophialophora*, *Alternaria*, and *Setophoma* (genera), were enriched in the flower fading stage and decreased in the full blooming stage, demonstrating the enrichment of the Pathotroph, Saprotroph, and Pathotroph–Saprotroph functions in the flower fading stage and their depletion in the full blooming stage. However, the total VOC and terpene contents were highest in the full blooming stage and lowest in the flower fading stage, which was opposite to the α diversity of the endophytic fungal community and the dominant fungi during flowering development. Linalool, dihydro-β-ionone, and *trans*-linalool oxide(furan) were key factors affecting the endophytic fungal community composition. Furthermore, dihydro-β-ionone played an extremely important role in inhibiting endophytic fungi in the full blooming stage. Based on the above results, it is believed that VOCs, especially terpenes, changed the endophytic fungal community composition in the flowers of *O. fragrans* ‘Rixiang Gui’. These findings improve the understanding of the interaction between endophytic fungi and VOCs in flowers and provide new insight into the mechanism of flower development.

## 1. Introduction

Endophytic fungi with high biodiversity are widely distributed in nature, where they are normally found within plant tissues. These fungi play an important role in plant development, the composition of the plant community, and the cycle of matter. Generally, endophytic fungi can promote plant growth or alleviate environmental stresses on plants, such as salinization, drought, and insect and pathogen infestation, through regulating plant hormones, inducing secondary metabolite production, and even producing secondary metabolites similar to those produced by plants themselves [1,2]. In turn, host plants exert selective pressure on endophytes and influence the composition of endophytic fungal communities [3,4]. Different plant organs produce diverse secondary metabolites in different developmental stages, such as flavonoids, triterpenoids, and polyphenols. These secondary metabolites can affect the composition and function of the endophytic community [5,6]. Therefore, the underlying relationship between the secondary metabolites released by host plants and endophytic fungal communities deserves more attention.

Volatile organic compounds (VOCs) are special plant secondary metabolites with low molecular weights, low boiling points, and volatility that play a role in attracting pollinators, defending against microbial pathogens, and responding to abiotic or biotic stress [7,8]. However, the effects of VOCs on the assembly of endophytic fungal communities have attracted less attention. Flowers are important ornamental organs of plants and one of the most important sources for the release of VOCs. Floral VOCs may be closely related to the composition of the endophytic fungal community. Most VOCs, including terpenes, benzene, and aliphatic compounds, have antibacterial properties [9], which may regulate the interactions among microorganisms, thereby affecting the endophytic fungal community through reducing pathogenic microorganisms. Furthermore, VOCs can influence the composition of floral microbial communities through microbial transmission mediated by pollinators [10,11]. In addition, floral microbes can release VOCs themselves [12] or metabolize floral VOCs, which may serve as a C source for microbes, such as methanol [13], thereby affecting floral VOC emission. To date, studies in this field have primarily focused on the release of floral VOCs [14,15], the composition of the floral microbial community [16,17], or the relationship between floral VOCs and microbes attached to the surface of plants. For example, the release of terpenoid VOCs from *Arabidopsis* flowers affects the composition of microorganisms on the flower surface [18], and the variation in VOCs released from strawberry flowers will lead to the change in the interfloral microbial community [19]. Although epiphytic and endophytic fungi coexist within a few millimeters of each other, their communities are quite different [20,21]. Therefore, the rarely reported relationship between floral VOCs and endophytic fungi needs further study.

*Osmanthus fragrans* has been cultivated for thousands of years in China and is famous for its unique fragrance. Currently, there are more than 200 cultivars of *O. fragrans*, including ‘Rixiang gui’, which is famous for its remontancy and long ornamental period. Previous studies reported that floral VOCs among different cultivars of *O. fragrans* varied in types and contents [15,22]. Terpenes, such as linalool, ionone, and their derivatives, are the main components in the fragrance (i.e., VOCs) of *O. fragrans* [23]. These terpenes may affect the endophytic fungal community in *O. fragrans* flowers due to their antibacterial properties [24,25]. The linalool content in the VOCs of ‘Rixiang gui’ is significantly higher than that of other cultivars [22]. The type and content of VOCs change with the development of flowers [26,27]. Therefore, further study is required to determine whether the VOCs emitted from ‘Rixiang gui’ affect its composition and the diversity of the endophytic fungal community at different stages of flower development.

**Figure 1:**
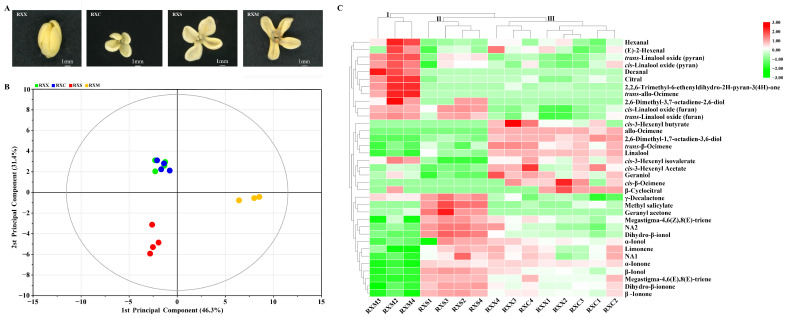
Gas chromatography–mass spectrometry (GC–MS) metabolomic analysis at four stages of flowering development. (**A**) Flowering stages of *Osmanthus fragrans* ‘Rixiang Gui’. (**B**) Score plot of all samples using principal component analysis (PCA). (**C**) Cluster heat map of all samples. Intensity values were adjusted using log transformation and then normalized.

To address the research gaps, metabolomics and high-throughput sequencing were used to study the relationship between VOCs and the endophytic fungal community at four stages of flower development in ‘Rixiang gui’, including the bud-eye stage (RXX), primary blooming stage (RXC), full blooming stage (RXS), and flower fading stage (RXM) (Figure 1A). Based on previous literature, it was hypothesized that the composition and diversity of the endophytic fungal community in the flowers of ‘Rixiang gui’ may change with flower development, which may be caused by different types and contents of VOCs at different stages of flower development. This study aimed to clarify the relationship between floral VOCs and endophytic fungi and how floral VOCs influenced the composition of the endophytic fungal community.

## 2. Results

### 2.1. Identification of O. fragrans ‘Rixiang Gui’ VOCs during Flowering

In this study, a total of 35 metabolites were identified at four stages of flower development, including terpenes, esters, aldehydes, and other compounds (Figure S1 and Table S1). Terpenes made up approximately 66% of the total metabolites (Figure S2A). In addition, the contents of total VOCs and terpenes in the RXS stage were the highest among all stages, followed by the RXC stage, and the contents of total VOCs and terpenes were the lowest in the RXM stage (Figure S2B). The contents of esters and other compounds were the highest in the RXS stage; however, the aldehydes were enriched in the RXM stage.

According to the results of principal component analysis (PCA), the two principal components explained 77.7% of the total variance with high predictive ability (Q^2^ = 68.1%), indicating that this model had good fit and predictability (Figure 1B). In the score plot, the samples in the RXM stage could be separated from samples in other stages by the first principal component, which suggested that the VOCs showed distinct differences between the RXM stage and the other three stages. The samples in the RXS and RXM stages could also be separated from those in RXX and RXC stages by the second principal component. However, the samples in the RXX and RXC stages were still stably mixed, implying that the RXX and RXC stages had similar aroma compounds. The results of hierarchal clustering were consistent with those of PCA and showed that all the samples were clustered into three categories (Figure 1C). Specifically, the VOCs in the RXM stage were clustered into one category (cluster I), the VOCs in the RXS stage were clustered into another (cluster II), and the VOCs in the RXX and RXC stages were clustered into a third category (cluster III). In the pattern of VOC release, some VOCs—including linalool oxides—were significantly enriched in the RXM stage, while other VOCs—including α/β-ionone and dihydro-β-ionone—were significantly accumulated at the RXS stage. The remaining VOCs, including linalool, ocimene, and others, were significantly enriched in the RXX and RXC stages.

### 2.2. Differential Release of VOCs at Four Stages of Flowering Development

**Figure 2:**
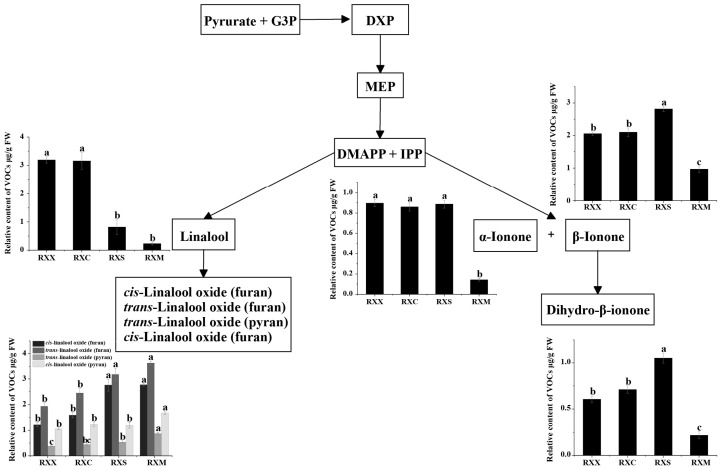
Dynamic changes in linalool, ionone, and their derivatives during the flowering process of *Osmanthus fragrans* ‘Rixiang Gui’. Data are shown as the mean value ± standard error (*n* = 4, except RXM which was 3 biological replicates). Different lowercase letters (a, b, c) above columns indicate significant differences at *p* < 0.05 according to Duncan’s test.

In the orthogonal partial least squares discrimination analysis (OPLS-DA) models (Figure S3A), the samples in the RXC and RXS stages were clearly separated, and similar results were obtained between the RXS and RXM stages. Notably, there was some overlap between the RXX and RXC stages in the PCA plots, but significant differences were observed in the OPLS-DA models, with the samples of the RXX stage grouped on the left, while the samples of the RXC stage were grouped on the right, indicating that the RXX and RXC stages could be distinguished. Moreover, for the RXX and RXC stages, fifteen VOCs (including ten terpenes) were the differential metabolites based on the variable influence on projection (VIP) value > 1. Nineteen VOCs (including thirteen terpenes) were identified as the differential metabolites in the RXC stage compared to the RXS stage, and there were nineteen differential VOCs (including twelve terpenes) in the RXS stage compared to the RXM stage. This study further quantitatively profiled these differential metabolites, and eight terpenes were screened based on their higher content, including *cis*/*trans*-linalool oxide (furan/pyran), linalool, α-ionone, dihydro-β-ionone, and β-ionone (Figure S3B). This implied that the differences in linalool, ionone, and their derivatives largely affected the scent of *O. fragrans* ‘Rixiang Gui’ at different flowering stages, and these VOCs could be regarded as representative VOCs at the four stages of flowering development. Therefore, this study further analyzed the trends in these VOCs. The content of linalool significantly decreased, while its derivatives, including *trans*-linalool oxide (furan), *cis*-linalool oxide (furan), *trans*-linalool oxide (pyran), and *cis*-linalool oxide (pyran), accumulated significantly with flowering time. Furthermore, the contents of β-ionone and dihydro-β-ionone initially increased and reached the peak at the RXS stage, subsequently decreasing, whereas α-ionone significantly decreased only at the RXM stage (Figure 2).

### 2.3. Alpha Diversity and Community Composition of Endophytic Fungi in O. fragrans ‘Rixiang Gui’ Flowers

**Figure 3:**
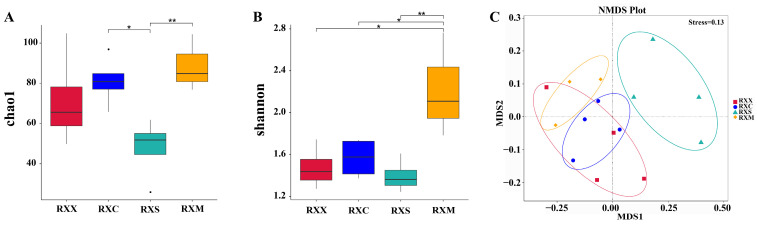
Alpha diversity ((**A**), Chao1 index; (**B**), Shannon index) and community composition ((**C**), non-metric multidimensional scaling (NMDS)) of endophytic fungi based on unweighted UniFrac distances at different stages of flowering development. Asterisks on the box-plots indicate statistically significant differences (* *p* < 0.05, ** *p* < 0.01).

A total of 1,204,462 high-quality reads and 289 amplicon sequence variants (ASVs) were obtained, and the rarefaction curves of all samples reached a saturation plateau, showing that the sequencing depth was sufficient for further analysis (Figure S4). The Chao1 and Shannon indices of endophytic fungi in flowers were the lowest at the RXS stage and were significantly higher at the RXM stage than at the RXS stage (*p* < 0.05) (Figure 3A,B). The results of non-metric multidimensional scaling (NMDS) and Adonis analysis showed that the community composition of endophytic fungi at the RXS stage was significantly different from that at other stages (Figure 3C and Table S2).

**Figure 4:**
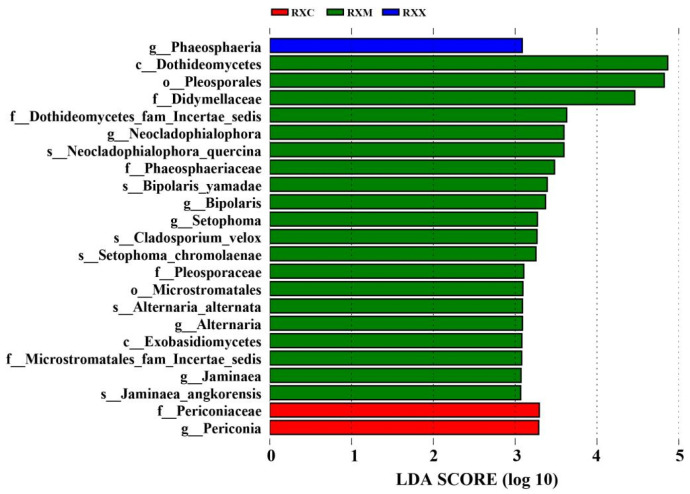
Linear discriminant analysis (LDA) scores observed for individual taxa in fungi surpassing the LDA effect size (LEfSe) significance threshold (LDA = 3, *p* < 0.05) among different stages of flowering development.

The Ascomycota and Basidiomycota phyla were observed. At the class level, the relative abundances of Dothideomycetes and Exobasidiomycetes at the RXM stage increased significantly compared with their abundances at the RXS stage (*p* < 0.05) (Figure S5A and Table S3). At the order level, the relative abundances of Pleosporales and Microstromatales at the RXM stage were significantly higher than at the RXS stage (*p* < 0.05), and at the RXX stage, the abundance of Trichosphaeriales was significantly higher than that at the RXS stage (*p* < 0.05) (Figure S5B and Table S4). At the genus level (Figure S5C and Table S5), the relative abundances of *Neocladophialophora*, *Alternaria*, *Setophoma*, *Jaminaea*, *Sclerostagonospora*, and *Phaeosphaeria* were significantly higher at the RXM stage than at the RXS stage (*p* < 0.05), and the relative abundances of *Nigrospora* and *Periconia* were also significantly higher at the RXC stage than at the RXS stage (*p* < 0.05). The relative abundance of *Toxicocladosporium* at the RXM stage increased significantly compared with the other three stages (*p* < 0.05). In addition, linear discriminant analysis effect size (LEfSe) analysis identified 23 endophytic fungi biomarkers. Among them, the genus *Phaeosphaeria* could be used as the biomarker of the RXX stage, and the family Periconiaceae and genus *Periconia* could be used as the biomarkers of the RXC stage. At the RXM stage, there were abundant clades, such as the class Dothideomycetes, the order Pleosporales, and the family Didymellaceae (Figure 4).

### 2.4. Functional Prediction of the Endophytic Fungi Community at Different Stages of Flowering Development

**Figure 5:**
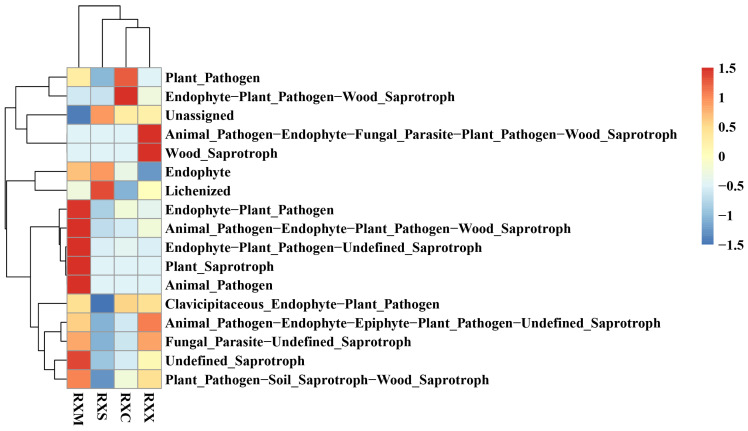
Functional profiles predicted by FUNGuild for endophytic fungi at four stages of flowering development in *Osmanthus fragrans* ‘Rixiang Gui’.

According to functional predictions and clustering (Table S6 and Figure 5), the RXC stage and RXX stage were grouped into one cluster, while the RXS and RXM stages were widely separated from the RXC and RXX stages. In addition, most Pathotroph, Saprotroph, and Pathotroph–Saprotroph functional profiles were enriched at the RXM stage and depleted at the RXS stage; for example, the Endophyte–Plant_Pathogen–Undefined_Saprotroph, Plant_Saprotroph, Animal_Pathogen, Endophyte–Plant_Pathogen, Animal_Pathogen–Endophyte–Plant_Pathogen–Wood_Saprotroph, and Undefined_Saprotroph functional profiles. Furthermore, Animal_Pathogen–Endophyte–Fungal_Parasite–Plant_Pathogen–Wood_Saprotroph, and Wood_Saprotroph functional profiles were enriched at the RXX stage, while Plant_Pathogen and Endophyte–Plant_Pathogen–Wood_Saprotroph functional profiles were enriched at the RXC stage.

### 2.5. Linkages between VOCs and Endophytic Fungal Communities in O. fragrans ‘Rixiang Gui’ Flowers

**Figure 6:**
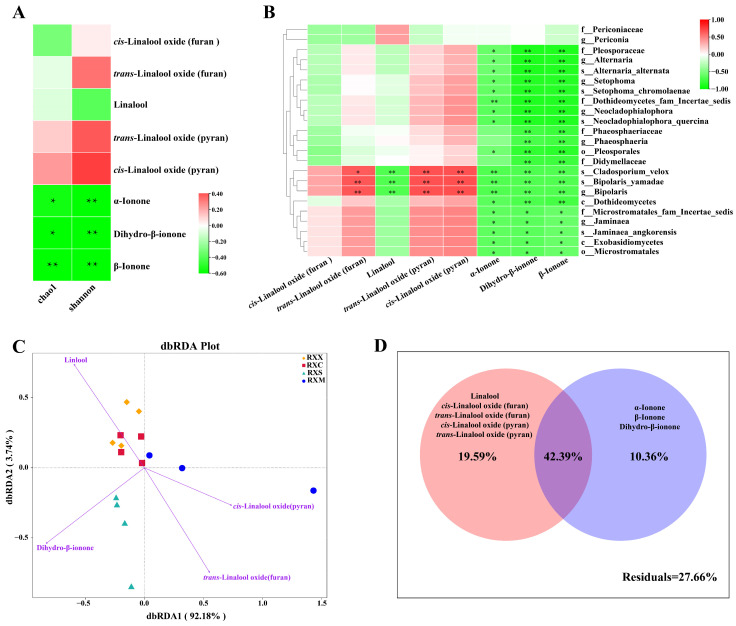
The relationship of the representative volatile organic compounds (VOCs) with the endophytic fungal community in *Osmanthus fragrans* ‘Rixiang Gui’ flowers. (**A**) Correlation analysis of the representative VOCs with alpha diversity indices of endophytic fungal communities. (**B**) Correlation analysis of the representative VOCs with 23 endophytic fungi biomarkers. (**C**) Distance-based redundancy analysis (dbRDA). (**D**) Variance partitioning analysis (VPA). Asterisks on the heat maps indicate statistically significant differences (* *p* < 0.05, ** *p* < 0.01).

The relationship between the endophytic fungal community and the representative VOCs was further analyzed. Spearman correlation analysis showed that α-ionone, β-ionone, and dihydro-β-ionone were negatively correlated with the Chao1 and Shannon indices (*p* < 0.05, Figure 6A). In addition, most of the biomarkers (91% of observed biomarkers) were negatively correlated with three ionone derivatives (α-ionone, β-ionone, and dihydro-β-ionone), such as classes Dothideomycetes and Exobasidiomycetes, orders Pleosporales and Microstromatales, and genera *Alternaria*, *Setophoma*, *Neocladophialophora*, and *Jaminaea* (*p* < 0.05, Figure 6B). Furthermore, the genus Bipolaris was significantly positively correlated with three linalool derivatives (*trans*-linalool oxide (furan) and *cis*/*trans*-linalool oxide (pyran)) and significantly negatively correlated with linalool.

Before distance-based redundancy analysis (dbRDA), linalool, dihydro-β-ionone, *cis*-linalool oxide(pyran), and *trans*-linalool oxide(furan) with variance inflation factor (VIF) < 20 were screened via VIF. The results of dbRDA showed that linalool, dihydro-β-ionone, and *trans*-linalool oxide(furan) were key factors affecting the community composition of endophytic fungi (*p* < 0.01, Table S7). Linalool played an important role in structuring the endophytic fungal community at the RXX and RXC stages, while dihydro-β-ionone played a significant part in structuring the endophytic fungal community at the RXS stage. In addition, *cis*-linalool oxide(pyran) and *trans*-linalool oxide(furan) were positively correlated with the community composition of endophytic fungi at the RXM stage (Figure 6C).

Variance partitioning analysis (VPA) showed that the variables linalool (including its derivatives) and ionone (including α-ionone, β-ionone, and dihydro-β-ionone) explained 61.98% and 52.75% of the variation in the endophytic fungal community composition, respectively. Linalool (including its derivatives) had stronger effects on the endophytic fungal community composition than ionone (including α-ionone, β-ionone, and dihydro-β-ionone) (Figure 6D).

## 3. Discussion

Geographical location of plant growth significantly affects its VOCs. For example, Yun et al. analyzed 306 black tea samples from 10 different geographical locations and found significant differences in VOCs from different locations [28]. Therefore, the same location was chosen for sampling in order to avoid environmental interference. The results indicated that the floral VOCs of *O. fragrans* ‘Rixiang Gui’ were affected by the stage of flower development, and the types and content of VOCs were the main cause of the difference in aroma between different stages. Previous studies have shown that linalool, ionone, and their derivatives are the main VOCs of *O. fragrans* [15,23], and the VOCs released from *O. fragrans* change with the development of flowers [26,27]. This finding was consistent with the present study, in which the highest VOC content was released at the RXS stage and the lowest VOC content was detected at the RXM stage.

Plant development plays an important role in structuring the symbiotic microbial community [29,30]. The results of the present study showed that the composition and α diversity of the endophytic fungal community in *O. fragrans* ‘Rixiang Gui’ shifted with different stages of flowering development. However, the shift did not represent linear succession over time. Generally, the main function of floral VOCs is to attract pollinators [31,32], and pollinators may influence the spread of microorganisms and thus the composition of microbial communities in flowers [10,11]. In the present study, the change in floral VOC content at different stages of flowering development in *O. fragrans* ‘Rixiang Gui’ was opposite to that of α diversity in the endophytic fungal community and that of the relative abundance of some dominant endophytic fungi (such as the class Dothideomycetes, the order Pleosporales, and the genera *Neocladophialophora*, *Alternaria*, and *Setophoma*). This finding indicates that VOCs may affect the endophytic fungal community composition. Previous studies have reported that plant VOCs can also act as antibiotics to resist the invasion of pathogens [33] and play an important role in structuring the microbes inhabiting plant surfaces, including stems, leaves, and flowers [13,34,35]. For example, the flowers of *Arabidopsis* can release the volatile sesquiterpene (E)-β-caryophyllene against pathogens [33]. The composition of the microbial community attached to flowers and leaves in *Saponaria officinalis* and *Lotus corniculatus* differs owing to different types and contents of VOCs released from different organs. The low α diversity of the epiphytic bacterial community in flowers is caused by the strong antibacterial effect of VOCs released from flowers [34]. Generally, host plants obtain their endophytic fungi vertically through their parents [36], but endophytic fungi can also be acquired via horizontal transmission, and most of these fungi may grow on the plant surface before penetrating plant tissues [1,37,38]. Therefore, this study speculated that floral VOCs may be the main factors driving the community composition of endophytic fungi under temporal alternations. In addition, according to functional prediction, Pathotroph and Saprotroph functions were enriched at the RXM stage and depleted at the RXS stage, which was opposite to the VOC content at the RXM and RXS stages. This indicated that blooming flowers could release VOCs inhibiting the growth of pathogens, while the decaying flowers with less VOCs release could serve as a C source for Saprophytic trophic fungi and pathogens and, in turn, accelerate the decay of the host flowers [39].

Terpenes, especially linalool, dihydro-β-ionone, and *trans*-linalool oxide(furan), were found to be key factors affecting the community composition of endophytic fungi at different stages of flowering development in *O. fragrans* ‘Rixiang Gui’. In contrast, the floral VOCs of strawberry, especially terpenes (α-pinene and β-pinene isomers), were found to significantly affect the changes in the interfloral microbial community [19]. This suggests that the VOCs that affect microbial communities differ among plant species. Previous research reported that essential oil extracted from the flower of *O. fragrans* and its main components, ionone and linalool oxide (furan/pyran), exhibited antimicrobial activity [40]. However, the present study found that *trans*-linalool oxide (furan/pyran) and *cis*-linalool oxide (pyran) were positively correlated with most differential indicator species and the Shannon index, whereas linalool was negatively correlated with these differential indicator species and the Shannon index (Figure 6). Boachon et al. found that the expression of *CYP706A3* (a cytochrome P450 coding gene) inhibited the release of sesquiterpenes and monoterpenes in the flowers of *Arabidopsis*, thereby increasing the diversity of the microbial community [18]. The cytochrome P450 genes were found to catalyze the synthesis of linalool oxide from linalool [41], indicating that the antibacterial ability of linalool oxide was weaker than that of linalool. In the present study, the content of linalool was higher in the early stage of flowering and significantly reduced in the later stage, while its derivatives accumulated significantly with the development of flowers (Figure 2). During this process, the synthesis of linalool oxide was catalyzed by *CYP450* genes, and the release of linalool was inhibited, thus lessening its inhibitory effect on endophytic fungi.

Generally, the endophytic fungi of plants can also produce VOCs. For example, *Talaromyces wortmannii* can produce β-caryphyllene [42]. Similarly, the VOCs released from *O. fragrans* ‘Rixiang Gui’ may contain VOCs produced by endophytic fungi. To some extent, it is difficult to precisely distinguish the origin of VOCs. To date, various bioactive terpenoids produced by endophytic fungi have been reported, including monoterpenoids, sesquiterpenoids, diterpenoids, sesterterpenoids, triterpenoids, and meroterpenoids [43]. However, the results of the present study showed that the trends of linalool and ionone released from ‘Rixiang Gui’ during flowering development were opposite to those of endophytic fungi, which corresponded to the antifungal properties of linalool and ionone [44,45,46]. Therefore, it is believed that the VOCs detected in this study were primarily released from flowers. In addition, the floral VOCs of *O. fragrans* are also influenced by other factors such as geographical environment (including temperature [47] and light [48]). Therefore, further study could be conducted to explore the role of the geographical environment in shaping the host VOCs and endophytic fungi of *O. fragrans* in the future.

## 4. Materials and Methods

### 4.1. Experimental Design

‘Rixiang gui’, an Asiaticus cultivar of *O. fragrans* that blooms all year round with a light aroma and white-yellow flowers, was used in this study. Four 30-year-old, healthy ‘Rixiang gui’ trees were selected with the same habitat and size in the campus of Nanjing Forestry University. Flowers at different stages of flower development, i.e., RXX, RXC, RXS, and RXM stages, were collected on sunny mornings (9:00–10:00 a.m.) from 8 to 12 October 2021. During each sampling, flowers from different directions on the tree were collected and mixed to form one sample, which was immediately put into an icebox and brought to the laboratory. Each sample was divided into two parts: (1) immediate VOC determination via gas chromatography–mass spectrometry (GC–MS) and (2) immediate sterilization with 75% ethanol, flash-freezing with liquid nitrogen, and storing the frozen sample in the refrigerator at −80 °C for the subsequent DNA extraction of endophytic fungi in the flowers.

### 4.2. Detection of VOCs

The floral VOCs were collected using headspace solid-phase microextraction (HS-SPME) and then determined using GC-MS. The samples of fresh flowers were ground in liquid nitrogen, and 0.3 g of the powder was weighed and transferred to a 10 mL headspace vial containing 2 mL NaCl saturated solution. Each vial was placed at 25 ± 5 °C for 30 min, and then a 65 µm polydimethylsiloxane (PDMS)/divinylbenzene (DVB) fiber (Supelco Co., Bellefonte, PA, USA) was exposed to the headspace of the capped vials for 35 min at 45 °C for extraction. Trace DSQ GC-MS (Thermo Fisher Scientific, Waltham, MA, USA) was immediately used at 250 °C to analyze the desorbed floral VOCs. Floral VOCs were separated on a 30 m × 0.25 mm × 0.25 mm TR-5MS capillary column (Supelco Co., Bellefonte, PA, USA), and the column oven temperature was programmed at 60 °C for 2 min, increasing at 5 °C/min to 150 °C, then increasing at 10 °C/min to reach 250 °C, and held for 1 min. Helium was used as the carrier gas at a linear velocity of 1.0 mL/min, and the injection port temperature was maintained at 250 °C. The mass spectrometry conditions were ionization energy of 70 eV and a scanning mass range of 33–450 *m*/*z*. Volatile compounds were first matched by mass spectra using the NIST98 database through ChemStation (Agilent Technologies, Santa Clara, CA, USA). A series of n-alkanes (C7–C30) (Sigma, St. Louis, MO, USA) were detected using GC–MS to obtain the linear retention indices (RIs) of VOCs. Subsequently, these RI values were compared with published linear retention indices (NIST Chemistry WebBook, SRD 69). Ethyl decanoate was used as an internal standard, and the normalization of peak areas was used to calculate the quantities of floral VOCs as described: content of each component (μg/g) = [(peak area of each component × content of internal standard)/peak area of internal standard]/sample weight.

### 4.3. DNA Extraction, Sequencing, Library Construction, and Processing

The DNA of endophytic fungi in flowers was extracted using the CTAB method. The ITS2 region was amplified using a specific primer pair (ITS5-1737F: 5′-GGAAGTAAAAGTCGTAACAAGG-3′, ITS2-2043R: 5′-GCTG CGTTCTTCATCGATGC-3′). The polymerase chain reaction (PCR) products were mixed in equidensity ratios and purified. Sequencing libraries were generated using the Illumina TruSeq DNA PCR-Free Library Preparation Kit (Illumina, San Diego, CA, USA). The library quality was assessed on a Qubit@ 2.0 Fluorometer (Thermo Fisher Scientific, Waltham, MA, USA) and an Agilent Bioanalyzer 2100 system. The library was sequenced on the Illumina NovaSeq platform using 250 bp paired-end sequencing at Novogene Bioinformatics Technology Co., Ltd. (Beijing, China).

Paired-end reads were merged using FLASH (Version 1.2.11) to obtain raw tags. After quality filtering, splicing, and chimera removal, the effective tags were obtained and denoised using the DADA2 module in the QIIME2 software (Version QIIME2-202006), after which the ASVs were obtained and ASVs with abundances less than 5 were filtered out. A pre-trained naive Bayes classifier was used to assign the taxonomy of the ASVs, and the classification information was annotated based on the UNITEv8.2 databases.

### 4.4. Statistical Analyses

Statistical analyses were performed using SPSS software version 20.0. Heat maps were generated using TBtools software (Version v1.09876). To clarify the influence of each flowering stage on the VOCs of *O. fragrans* ‘Rixiang Gui’, PCA of four flowering stages and OPLS-DA of RXX vs. RXC, RXC vs. RXS, and RXS vs. RXM were performed using SIMCA-13.0 software (Version 13.0.3), and the differential metabolites were screened based on VIP value analysis (VIP > 1, *p* < 0.05). Bar graphs of the different VOCs were created using Origin Pro 2018. Spearman correlation analysis was used to evaluate the correlation between α diversity (Chao1 and Shannon indices), biomarkers of the endophytic fungal community, and floral VOCs.

Based on the ASV information, the rarefaction curves and Shannon and Chao1 indices were calculated using Mothur v. 1.30.1. The similarities between the fungal communities during the four stages of flower development were determined via NMDS based on the unweighted UniFrac distance. dbRDA between differential metabolites and fungal community composition at the ASV level were constructed using R software (Version 3.4.0). LEfSe was performed to identify significantly abundant fungal taxa (LDA score > 3.5; *p* < 0.05) among different stages of flower development. VPA was conducted to quantify the species distributions according to linalool (including its derivatives) and ionone (including α-ionone, β-ionone, and dihydro-β-ionone). Function prediction was performed using FUNGuild (https://github.com/UMNFuN/ FUNGuild, accessed on 27 September 2022).

## 5. Conclusions

**Figure 7:**
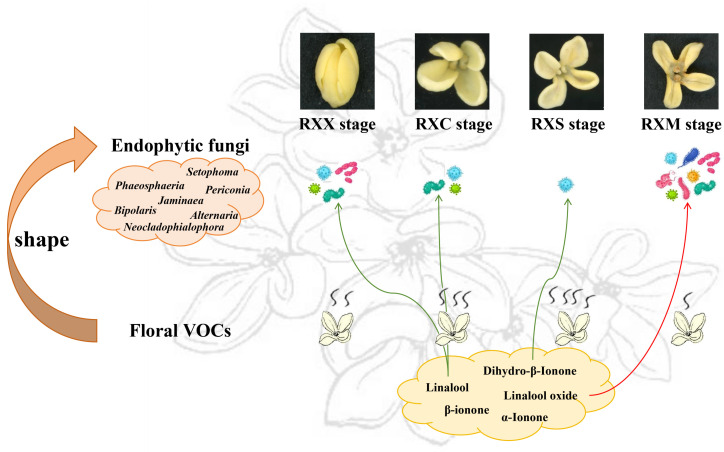
A summary plot showing the influences of floral VOCs on endophytic fungal community. Curve arrows of different colors indicate the key floral VOCs at different flowering stages influencing the endophytic fungal community. Curved arrows are shown in red for a positive correlation or in green for a negative correlation.

The composition of the endophytic fungal community and floral VOCs shifted along with flowering development. The dominant fungi, such as Dothideomycetes (class), Pleosporales (order), and *Neocladophialophora*, *Alternaria*, and *Setophoma* (genera), were enriched in the flower fading stage and decreased in the full blooming stage, demonstrating the enrichment of the Pathotroph, Saprotroph, and Pathotroph–Saprotroph functions in the flower fading stage and their depletion in the full blooming stage. However, the variations in total VOC and terpene contents were opposite to those in the relative abundance of dominant fungi during flowering development. Furthermore, linalool, dihydro-β-ionone, and *trans*-linalool oxide (furan) were key factors affecting the community composition of endophytic fungi. Overall, with the flowering development, the variation in floral VOCs was a vital factor affecting the composition of endophytic fungal community in flowers of ‘Rixiang Gui’ (Figure 7). Our study provided new insights into the floral VOCs mediating the variation in endophytic fungal communities during the flowering process.

## Data Availability

All data in this study can be found in the manuscript, supplemental materials, and publicly available repositories. Sequence data from this study were submitted to the NCBI Sequence Read Archive under accession number SRP438769.

## Supplementary Materials

The following supporting information can be downloaded at: https://www.mdpi.com/article/10.3390/ijms25020857/s1.
