# Supplementary material for: Floral Volatile Organic Compounds Change the Composition and Function of the Endophytic Fungal Community in the Flowers of Osmanthus fragrans
Source: Int J Mol Sci. 2024 Jan 10;25(2):857. doi: 10.3390/ijms25020857 (PMC10815108; doi:10.3390/ijms25020857)
Supplement: Supplementary file 1 [file ijms-25-00857-s001.zip › Supplementary Figures.pdf]

Supplementary Figures

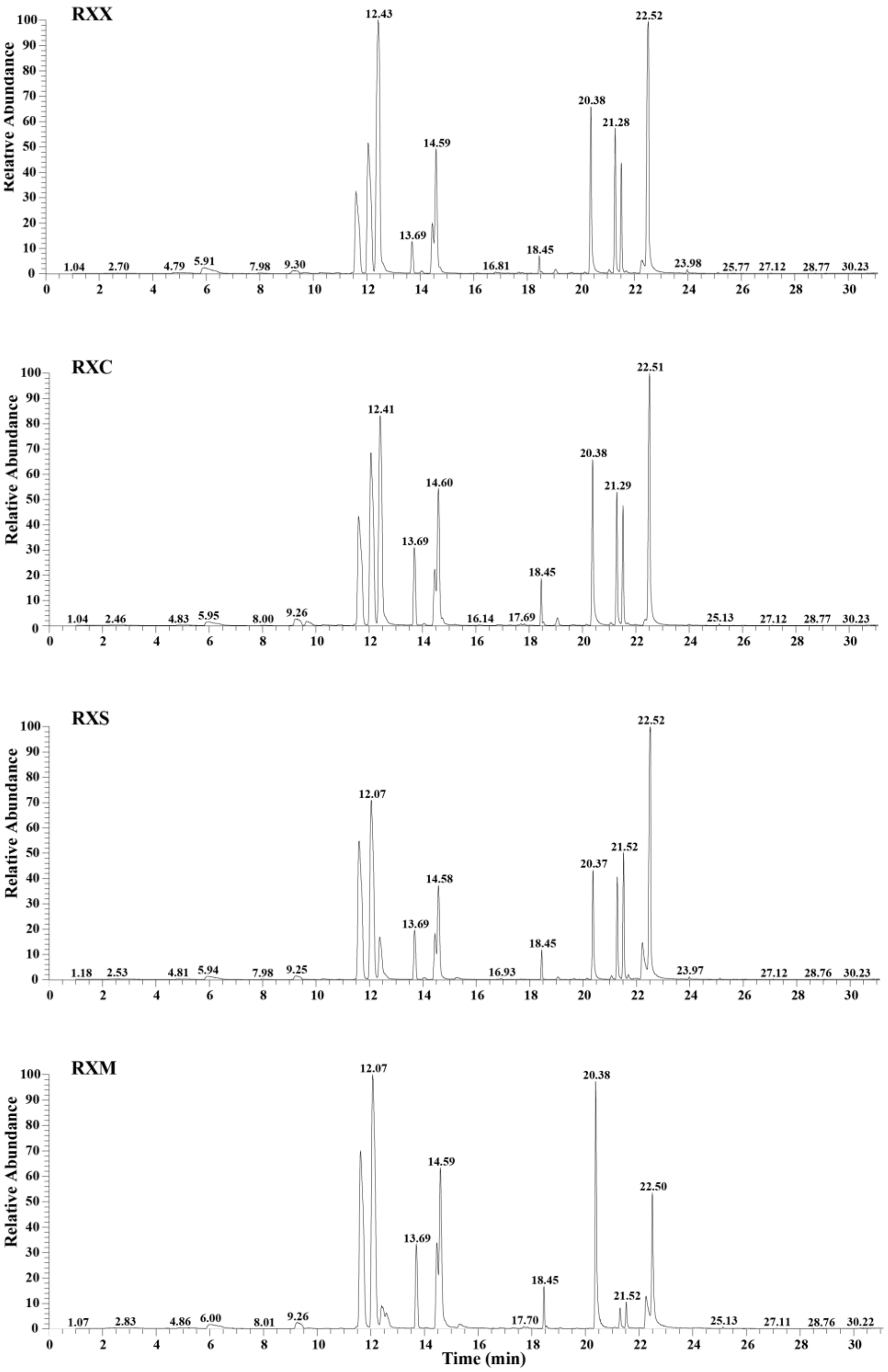

Figure S1. Typical chromatograms among four flowering stages of *Osmanthus fragrans* 'Rixiang Gui'.

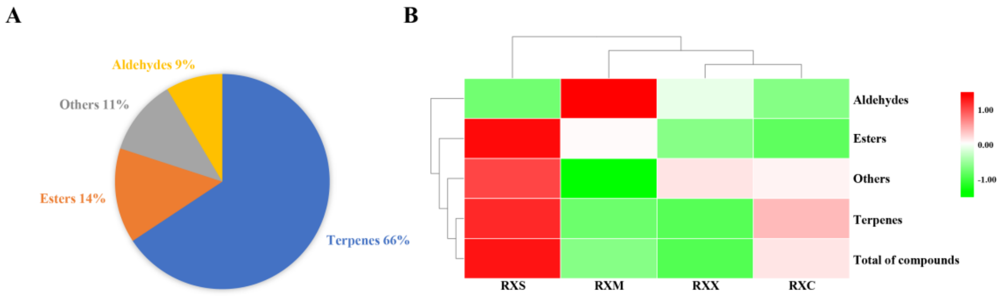

Figure S2. Volatile organic compounds (VOCs) analysis of *Osmanthus fragrans* 'Rixiang Gui'. (A) Classification and proportion of total VOCs detected in sweet osmanthus. (B) Heat map of the changes in the VOC types for four flowering stages.

3

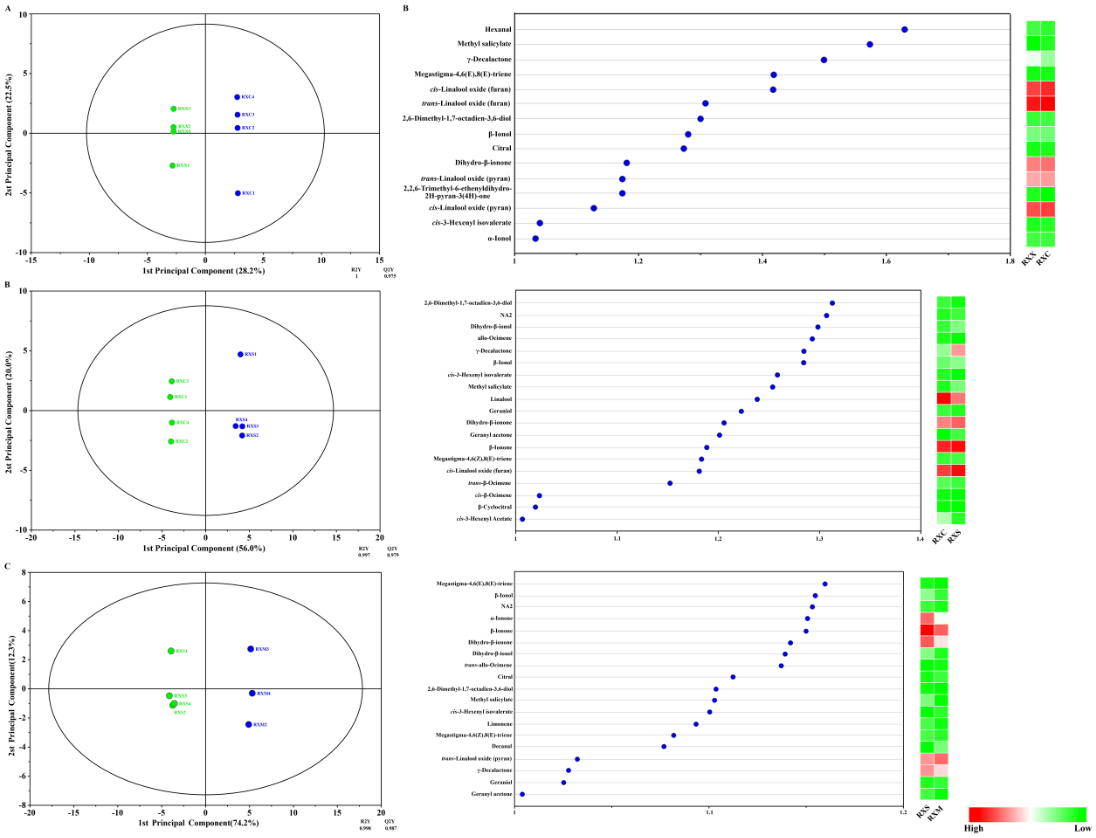

Figure S3. The orthogonal partial least squares discrimination analysis (OPLS-DA). (A) Score scatter plot of OPLS-DA models. (B) Variable importance projection of OPLS-DA and heatmaps of the differential metabolites.

4

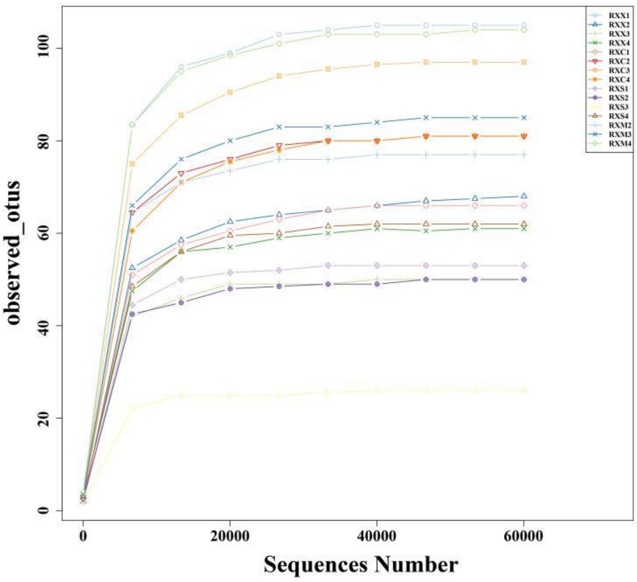

Figure S4. Rarefaction curves.

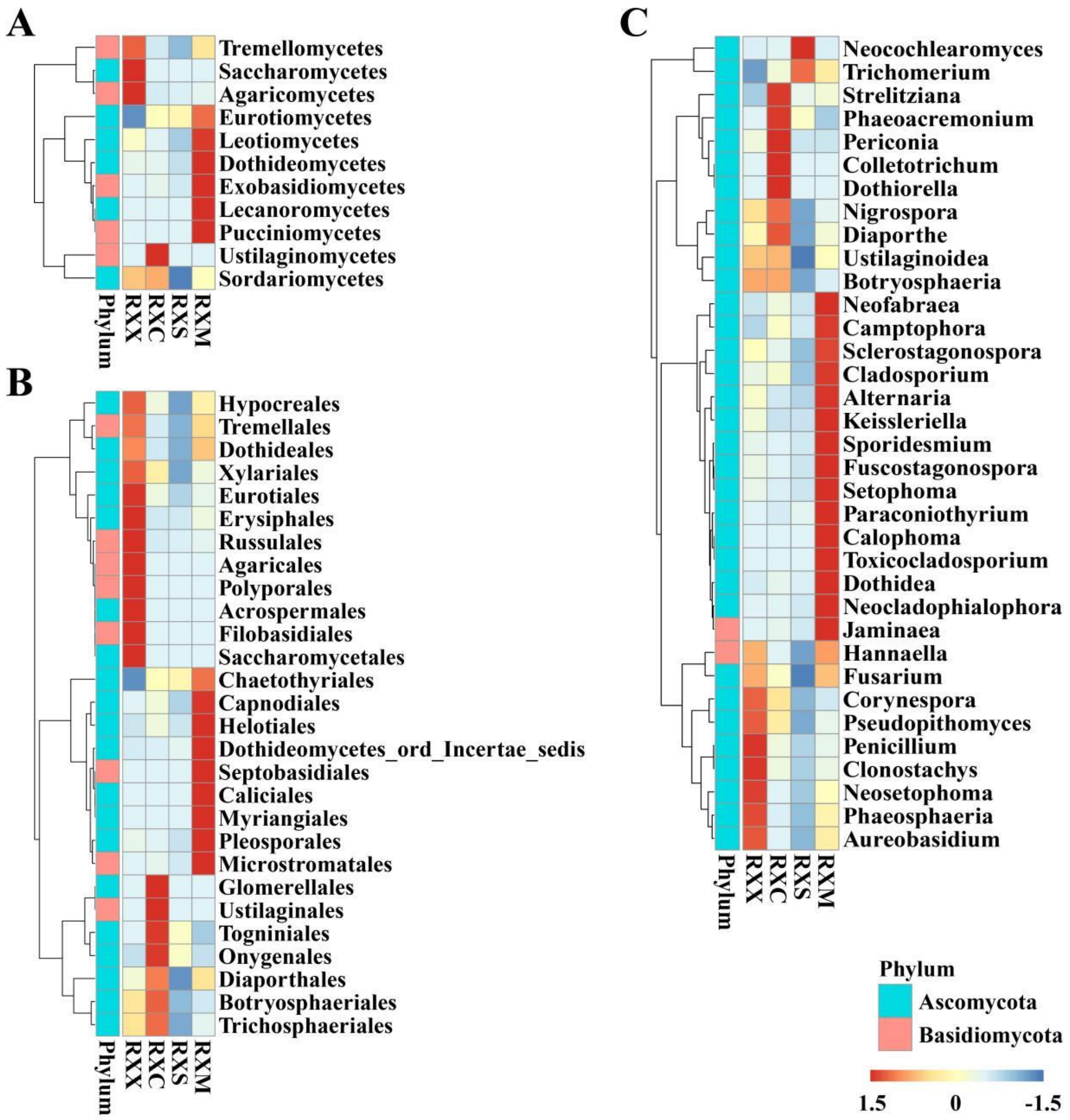

Figure S5. Heat map of the relative abundance of the endophytic fungi community from flowers of four flowering stages. (A) Endophytic fungal relative abundance at the class level, (B) endophytic fungal relative abundance at the order level, and (C) endophytic fungal relative abundance at the genus level. Flower square colors shift from dark red to blue, with darker red colors indicating higher abundance and darker blue colors indicating lower abundance.

6
